# Checkpoint-Inhibitoren – Indikation und Verwendung bei Melanompatienten

**DOI:** 10.1007/s00393-020-00870-8

**Published:** 2020-09-14

**Authors:** C. Lamos, R. E. Hunger

**Affiliations:** Universitätsklinik für Dermatologie, Inselspital Bern, Freiburgstr. 34, 3010 Bern, Schweiz

**Keywords:** Melanomtherapie, Systemtherapie, PD-1-Inhibitor, CTLA-4-Blocker, „Immuno-oncology“, Melanoma therapy, System therapy, PD-1 inhibitor, CTLA-4 blocker, Immuno-oncology

## Abstract

Die gesamte Onkologie wurde durch die Einführung von Ipilimumab, einem Checkpoint-Inhibitor, im Jahr 2011 revolutioniert. Seitdem wurden weitere effektive Checkpoint-Inhibitoren, wie die PD-1-Antikörper Nivolumab und Pembrolizumab entwickelt. Die Ergebnisse sind bahnbrechend, insbesondere beim fortgeschrittenen malignen Melanom, welches bis vor Kurzem in den meisten Fällen nach wenigen Monaten zum Tode führte. Die Anwendung der Checkpoint-Inhibitoren wurde mit vielversprechenden Ergebnissen auf weitere Tumorentitäten ausgeweitet.

## Abstract (en)

Since its introduction in 2011 the checkpoint inhibitor ipilimumab, has revolutionized the whole of oncology. After this first major step more checkpoint inhibitors, such as the PD‑1 antibodies nivolumab and pembrolizumab have been developed. The results are groundbreaking, especially in advanced malignant melanoma, which only a few years ago led to certain death in most cases after only a few months. Currently, the application of checkpoint inhibitors has been extended to many more tumor entities, with very promising results.

Jährlich erkranken weltweit fast 200.000 Menschen an einem malignen Melanom. Die jährliche Inzidenz des malignen Melanoms in Europa liegt in den nördlichen Ländern bei 12–35/100.000 und in den mediterranen Ländern bei 3–5/100.000. Die höchste jährliche Inzidenz wurde für Australien und Neuseeland mit 50/100.000 Fällen ermittelt. Es wurde eine stetige Inzidenzsteigerung in den letzten 40 Jahren registriert. Zudem sieht man einen Trend der Stabilisierung der Mortalität, mit Ausnahme der Mortalität von älteren Männern, die weiterhin ansteigt [1].

Das mittlere Erkrankungsalter liegt für Frauen bei 60 und für Männer bei 64 Jahren. Bei Frauen zwischen 20 und 30 Jahren ist das Melanom in Deutschland der häufigste maligne Tumor [2].

Lokal begrenzte Melanome können mittels operativer Intervention kurativ behandelt werden. Dabei ist v. a. die Tumordicke nach Breslow bei der Erstdiagnose das wichtigste Prognosekriterium. Diese stellt die absolute Dicke des Tumorgewebes in Millimeter vom Stratum granulosum der Epidermis bis zum tiefsten noch nachweisbaren Tumorgewebe dar. Weitere wichtige Prognosekriterien sind Ulzeration und Lymphknotenbefall. Aktuell erfolgen die Klassifikation und Stadieneinteilung des malignen Melanoms anhand der achten Edition der AJCC-Klassifikation (Tab. 1, 2 und 3; [3]).

**Table 1:** 

T	Tumordicke	Ulzerationsstatus
Tx (Dicke des Primärtumors kann nicht bewertet werden)	Kann nicht bewertet werden	Kann nicht bewertet werden
T0 (Primärtumor kann nicht nachgewiesen werden)	Kann nicht bewertet werden	Kann nicht bewertet werden
Tis (Melanoma in situ)	Kann nicht bewertet werden	Kann nicht bewertet werden
T1a	<0,8 mm	Ohne Ulzeration
T1b	<0,8 mm	Mit Ulzeration
T1b	0,8–1,0 mm	Mit oder ohne Ulzeration
T2a	>1,0–2,0 mm	Ohne Ulzeration
T2b	>1,0–2,0 mm	Mit Ulzeration
T3a	>2,0–4,0 mm	Ohne Ulzeration
T3b	>2,0–4,0 mm	Mit Ulzeration
T4a	>4,0 mm	Ohne Ulzeration
T4b	>4,0 mm	Mit Ulzeration

**Table 2:** 

N	Anzahl der metastasierten Lymphknoten	In-Transit‑, Satelliten- und/oder Mikrosatellitenmetastasen
N x	Lokale Lymphknoten nicht bewertet	Nein
N0	Keine lokalen Metastasen festgestellt	Nein
N1a	1 klinisch okkult	Nein
N1b	1 klinisch detektiert	Nein
N1c	Erkrankung ohne lokale Metastasen	Ja
N2a	2 oder 3 klinisch okkult	Nein
N2b	2 oder 3 und mindestens 1 davon klinisch detektiert	Nein
N2c	1 klinisch okkult oder detektiert	Ja
N3a	4 oder mehr klinisch okkult	Nein
N3b	4 oder mehr und 1 davon klinisch nachgewiesen oder verbackene Knoten	Nein
N3c	2 oder mehr klinisch okkult oder klinisch nachgewiesen und/oder Auftreten beliebig vieler verbackener Knoten	Ja

**Table 3:** 

T‑Klassifikation	N‑Klassifikation	M‑Klassifikation	Stadium
Tis	N0	M0	0
T1a	N0	M0	IA
T1b	N0	M0	IB
T2a	N0	M0	IB
T2b	N0	M0	IIA
T3a	N0	M0	IIA
T3b	N0	M0	IIB
T4a	N0	M0	IIB
T4b	N0	M0	IIC
T1a/b-T2a	N1a oder N2a	M0	IIIA
T0	N1b, N1c	M0	IIIB
T1a/b-T2a	N1b/c oder N2b	M0	IIIB
T2b/T3a	N1a–N2b	M0	IIIB
T0	N2b, N2c, N3b oder N3c	M0	IIIC
T1a-T3a	N2c oder N3a/b/c	M0	IIIC
T3b/T4a	Jedes N ≥ N1	M0	IIIC
T4b	N1a-N2c	M0	IIIC
T4b	N3a/b/c	M0	IIID
Jedes T	Jedes N	M1	IV

Bis vor wenigen Jahren war der Behandlungsanspruch bei fortgeschrittenen und metastasierten Melanomen palliativ, die mittlere Überlebenszeit lag bei 6 bis 12 Monaten. Standard waren Chemotherapien.

Eine Wende wurde durch die Einführung des Immuncheckpoint-Inhibitors Ipilimumab im Jahr 2011 eingeleitet – weg von klassischen Chemotherapien hin zu dem Konzept der Immuntherapie. Seit der Entwicklung von Ipilimumab, einem CTLA-4-Blocker (engl. für „cytotoxic T‑lymphocyte-associated protein 4“), sind neue hochwirksame Substanzen eingeführt worden, sowohl Checkpoint-Inhibitoren als auch zielgerichtete Kinasehemmer wie BRAF- und MEK-Inhibitoren. Checkpoint-Inhibitoren aktivieren die Tumorabwehr, in dem inhibitorische Interaktionen zwischen Antigen-präsentierenden Zellen und T‑Lymphozyten an den Interaktionsstrukturen, den sog. Checkpoints gehemmt (beispielsweise Anti-PD-1/PD-L1, Anti-CTLA-4) oder aktivierende Checkpoints stimuliert werden.

CTLA‑4 ist ein Mitglied der Immunglobulin-Superfamilie, welches unter anderem auf der Oberfläche von T‑Helferzellen und zytotoxischen T‑Zellen exprimiert wird. Für eine komplette T‑Zell-Aktivierung sind mindestens 2 Rezeptor-Liganden-Interaktionen notwendig. Die erste Interaktion findet zwischen dem spezifischen T‑Zell-Rezeptor und seinem Antigen statt, einem Peptid, welches über ein MHC-Molekül präsentiert wird. Für eine komplette T‑Zell-Aktivierung bedarf es aber eines zweiten Signals, welches sich auf der gleichen Antigen-präsentierenden Zelle wie der Peptid-MHC-Komplex befindet. Dieses Signal wird von kostimulierenden Molekülen (CD80 und CD86) an einen T‑Zell-Rezeptor, CD28, übertragen. Nur durch diese Interaktionen können spezifische T‑Zellen Effektorfunktionen erlangen und zu den Lokalisationen der Antigenexpression migrieren. Im Vergleich zu CD28 hat CTLA‑4 eine höhere Affinität zu CD80/CD86 und wirkt dabei antagonistisch. Über CTLA‑4 wird eine Proteinkinase aktiviert, die eine Proliferation der T‑Zelle hemmt. Gleichzeitig veranlasst eine Aktivierung der T‑Lymphozyten durch den T‑Zell-Rezeptor eine gesteigerte Expression von CTLA‑4 als Autoregulationsmechanismus. Über diesen Signalweg wird einer Überreaktion des Immunsystems entgegengewirkt. Eine Blockade von CTLA‑4 durch Ipilimumab löst somit diese physiologische „Bremse“ der T‑Zell-Aktivierung [4].

CTLA‑4 hat eine Rolle in der frühen T‑Zell Aktivierung, während der PD-1-Signalweg (engl. „programmed cell death 1“) eine Funktion in einer späteren Phase der Immunantwort hat. PD‑1 ist ein Transmembranprotein aus der Immunglobulin-Superfamilie, welches auf T‑, B‑ und natürlichen Killerzellen exprimiert wird. Es ist ein inhibitorisches Molekül, welches an PD-1-Ligand (PD-L1) und PD-2-Ligand (PD-L2) bindet. PD‑1 besitzt eine wichtige Rolle in der Regulation des Immunsystems. Die Interaktion von PD‑1 und PD-L1/L2 wirkt einer Autoimmunität entgegen, in dem sie die Apoptose von Antigen-spezifischen T‑Zellen in Lymphknoten fördert und die Apoptose in regulatorischen T‑Zellen reduziert. PD-L1 und PD-L2 werden auf einer Vielzahl von Körperzellen, inklusive Tumorzellen, exprimiert. PD‑1 wird durch persistierende Antigenexposition heraufreguliert, wie sie bei chronischen Infektionen oder Tumorerkrankungen auftritt. Expression von PD-L1 und PD-L2 wird durch inflammatorische Zytokine wie Interferon‑γ induziert. Die Interaktion von PD‑1 auf der T‑Zelle mit PD-L1/L2 auf der Tumorzelle führt zu einer Inhibition der T‑Zell-Aktivierung. Somit wird durch die PD-1-Blockade mit Pembrolizumab und Nivolumab eine Aktivierung des Immunsystems zur Tumorbekämpfung erreicht [5].

## Adjuvante Therapie des Melanoms mit Checkpoint-Inhibitoren

Die Therapie der Wahl früh diagnostizierter Melanome ist die chirurgische Entfernung. Diese ist in den meisten Fällen kurativ, wenn sie frühzeitig erfolgt. Bei einem Teil der Patienten kommt es allerdings zum Rezidiv mit disseminierter Erkrankung. Risikofaktoren des Primärtumors wie hohe vertikale Tumordicke (nach Breslow), Ulzeration sowie Befall der regionären Lymphknoten definieren Patientengruppen, die ein erhöhtes Risiko für einen Rezidiv der Erkrankung haben. Nach einer Ära der adjuvanten Interferontherapien für maligne Melanome im Stadium II und III mit enttäuschenden Langzeitergebnissen ist die Zeit revolutionärer adjuvanter Therapien angebrochen – einerseits die Immuntherapie mit den Checkpoint-Inhibitoren Pembrolizumab und Nivolumab, andererseits die zielgerichtete Therapie mit BRAF- und MEK-Inhibitoren (bei BRAF-V600-mutierten Melanomen). Direkte Vergleiche in Studien zwischen adjuvanter Therapie mit PD-1-Inhibitoren und dieser mit BRAF-/MEK-Inhibitoren sind bisher nicht erfolgt. Somit können bei BRAF-mutierten Patienten keine eindeutigen Empfehlungen bezüglich der adjuvanten Therapie gemacht werden. Es gilt, gemeinsam mit dem Patienten eine individuelle Therapieentscheidung zu treffen. Faktoren, die bei der Entscheidungsfindung aktuell zu beachten sind, wären Toxizitätsprofil, Applikationsmodus und Alter des Patienten.

### Adjuvante Therapie des malignen Melanoms mit PD-1-Inhibitoren

PD-1-Inhibitoren kommen adjuvant beim fortgeschrittenen malignen Melanom im Stadium III oder IV der Erkrankung zum Einsatz. Zugelassen sind die PD-1-Antikörper Pembrolizumab sowie Nivolumab, die sich nach aktuellem Kenntnisstand lediglich in ihrer Verabreichung unterscheiden: Pembrolizumab (200 mg intravenös) wird alle 3 Wochen verabreicht, während Nivolumab (240 mg intravenös) alle 2 Wochen verabreicht wird. Die adjuvante Therapie sollte 1 Jahr lang bis zu nicht tolerabler Toxizität oder einem Progress durchgeführt werden. Beide Substanzen können neuerdings auch in höheren Dosierungen und dafür größeren Intervallen verabreicht werden ohne Wirksamkeitsverlust oder höhere Toxizität (480 mg Nivolumab alle 4 Wochen und 400 mg Pembrolizumab alle 6 Wochen) [6, 7]. Die Substanzen sind EU-weit bereits in den höheren Dosierungen zugelassen.

Kürzlich konnte mit der CheckMate 238-Studie [8] die Überlegenheit der adjuvanten Therapie mit Nivolumab im Vergleich zu Ipilimumab in den Stadien IIIB/C und IV demonstriert werden. Im Nivolumab-Arm zeigte sich ein signifikant verbessertes rezidivfreies Überleben („relapse-free survival“ [RFS]): Hazard Ratio (HR) von 0,66, 70 % der Patienten rezidivfrei vs. 60 % nach 12 Monaten, 66 % vs. 53 % nach 18 Monaten und 63 % vs. 50 % nach 24 Monaten. Im September 2019 wurden zudem die Daten zum RFS nach 36 Monaten auf dem ESMO-Kongress in Barcelona vorgestellt: 58 % RFS mit Nivolumab vs. 45 % RFS mit Ipilimumab. Ein weiterer Vorteil der Therapie mit Nivolumab im Vergleich zu Ipilimumab stellt die deutlich geringere Toxizität mit weniger Grad 3–4 „adverse events“ dar: 14,4 % vs. 45,9 %.

Auch für Pembrolizumab adjuvant verglichen mit Placebo zeigte eine Studie [9] vielversprechende Ergebnisse. Eingeschlossen wurden Patienten im Stadium IIIA (nur Patienten mit einer Sentinelmetastasengröße von >1 mm), Stadium IIIB und Stadium IIIC. Nach einem medianen Follow-up von 15 Monaten war Pembrolizumab mit einem signifikant verlängerten RFS im Vergleich zu Placebo assoziiert: 1‑Jahres-RFS 75,4 % (95 %-KI 71,3–78,9) vs. 61 % (95 %-KI 56,5–65,1); HR für Rezidiv oder Tod 0,57 (98,4 %-KI 0,43–0,74), *p* < 0,001.

Gesamtüberlebensdaten sind aktuell weder für Nivolumab noch für Pembrolizumab verfügbar. Basierend auf diesen Daten zum RFS, kam es zur Zulassung beider Substanzen durch die EMA als adjuvante Therapie: Im August 2018 wurde Nivolumab und im Dezember 2018 Pembrolizumab zugelassen.

Es konnte gezeigt werden, dass Patienten im Stadium IIC der Erkrankung eine ebenso schlechte Prognose haben wie Patienten im Stadium IIIB. Das melanomspezifische Überleben für Stadium IIC beträgt 82 %, während es für Stadium IIIB 83 % beträgt [3]. Somit liegt es nahe, dass auch Stadium IIC-Patienten von einer adjuvanten Therapie profitieren könnten. Aktuell rekrutiert die KEYNOTE 716 (MK3475-716)-Studie Patienten mit reseziertem Melanom im Stadium IIB und Stadium IIC zur Untersuchung einer adjuvanten Therapie mit Pembrolizumab vs. Placebo. Es wird allerdings noch Jahre dauern bis Ergebnisse vorliegen.

### Adjuvante Immuntherapie des malignen Melanoms mit anderen Checkpoint-Inhibitoren

Ein anderer Checkpoint-Inhibitor, der bei Melanompatienten im Stadium III in der Adjuvans untersucht wurde, ist Ipilimumab. Es handelt sich um einen monoklonalen Antikörper, der das zytotoxische T‑Lymphozyten-assoziierte Antigen 4 (CTLA-4) blockiert, welches eine essenzielle Rolle in der Regulation der spezifischen Immunantwort spielt. Es wurde im Jahr 2011 als erster Checkpoint-Inhibitor zur Therapie des fortgeschrittenen malignen Melanoms zugelassen. In einer Studie zeigte es in der Adjuvans ein verbessertes RFS verglichen mit Placebo (3-Jahres-RFS 46,5 % vs. 34,8 %, *p* = 0,0013) [10]. Die 5‑Jahres-Gesamtüberlebensrate (OS) lag bei 65,4 % in der Ipilimumab-Gruppe vs. 54,4 % in der Placebogruppe (*p* = 0,001). Allerdings kam es nicht zu einer Zulassung durch die EMA (European Medicines Agency) aufgrund der ausgeprägten, teils irreversiblen Toxizitäten. Zudem führte die adjuvante Therapie mit Nivolumab oder Pembrolizumab zu einem vergleichsweise verlängerten RFS.

## Neue Therapiestrategien: neoadjuvante Therapie des fortgeschrittenen malignen Melanoms

Der Einsatz von Checkpoint-Inhibitoren im neoadjuvanten Setting erscheint vielversprechend. In einer Studie kam es bei 8 von 27 Patienten mit einer einzigen neoadjuvanten Gabe eines PD-1-Inhibitors zu einem kompletten oder sehr guten histologischen Ansprechen, zudem blieben alle Patienten progressionsfrei [11]. In einer weiteren randomisierten Phase-II-Studie wurden Patienten in einem Arm neoadjuvant mit einer Kombinationstherapie, bestehend aus Ipilimumab und Nivolumab, behandelt, während in einem weiteren Arm neoadjuvant mit Nivolumab-Monotherapie behandelt wurde. Die Patientengruppe mit der Kombinationstherapie erzielte hohe Ansprechraten (Gesamtansprechrate: 73 %, komplettes histologisches Ansprechen: 45 %). Allerdings waren die Toxizitäten nicht zu vernachlässigen (73 % Grad-3-Nebenwirkungen), während die Monotherapie eher zu einer bescheidenen Gesamtansprechrate von 25 % führte, mit einem kompletten histologischen Ansprechen von 25 %, allerdings auch mit niedrigeren Toxizitätsraten von 8 % Grad-3-Nebenwirkungen [12].

Neoadjuvante Immuntherapien erscheinen vielversprechend, obwohl diese anscheinend mit erhöhten Toxizitäten vergesellschaftet sind. So müssen zukünftige Studien noch klären, wann diese Strategie gegenüber einer adjuvanten Therapie bevorzugt werden sollte. Zurzeit werden nicht nur Checkpoint-Inhibitoren im neoadjuvanten Setting, sondern auch Kinaseinhibitoren untersucht.

## Therapie des malignen Melanoms im Stadium IV oder nichtresektablen Stadium III mit Checkpoint-Inhibitoren

Bis vor wenigen Jahren waren die einzigen Therapiemöglichkeiten des Melanoms im Stadium IV oder nichtresektablen Stadium III diverse Chemotherapieregime mit äußerst schlechten Prognoseaussichten für die Patienten. Die Einführung der Checkpoint-Inhibitoren, aber auch der BRAF- und MEK-Inhibitoren revolutionierte die Therapie des Melanoms. Die Patienten haben nun einen deutlichen Überlebensvorteil im Vergleich zu den Zeiten, als die einzigen Therapieoptionen traditionelle Chemotherapien waren.

Der aktuelle Erstlinientherapiestandard für das fortgeschrittene Melanom im nichtresektablen Stadium III und IV sind PD-1-Inhibitoren (Nivolumab oder Pembrolizumab) als Monotherapie oder in Kombination mit einem CTLA-4-Blocker (Ipilimumab). Beim BRAF-V600-mutierten Melanom kommen als weitere Möglichkeit zu den Checkpoint-Inhibitoren BRAF-Inhibitoren (Vemurafenib, Dabrafenib, Encorafenib) in Kombination mit MEK-Inhibitoren (Cobimetinib, Trametinib, Binimetinib) infrage. Ferner besteht bei nichtresektablem Stadium IIIB/C und Stadium IV M1a die Möglichkeit einer Therapie der von außen zugänglichen Metastasen mit einem injizierbaren gentechnisch veränderten onkolytischen Virus, Talimogene laherparepvec (T-VEC).

Aktuell stellt sich die Frage, welches die optimale Therapiesequenz ist. In den meisten Zentren erhalten Patienten im Stadium IV, die eine geringe Tumorlast haben, zuerst eine Therapie mit Checkpoint-Inhibitoren. Dies ist einerseits dadurch zu erklären, dass Checkpoint-Inhibitoren meistens eine längere Zeit bis zur Wirkung im Vergleich zu BRAF- und MEK-Inhibitoren brauchen. Andererseits erhofft man sich ein längeres Ansprechen auf die Therapie im Vergleich zu BRAF- und MEK-Inhibitoren. Patienten mit einer positiven BRAF-V600-Mutationsanalyse, die eine hohe Tumorlast haben, bei denen die Erkrankung eine wichtige Organfunktion bedroht und deren Lebenserwartung auf wenige Tage oder Wochen geschätzt wird, erhalten aber in den meisten Zentren in erster Linie eine zielgerichtete Kombinationstherapie mit einem BRAF/MEK-Inhibitor, da man sich die (bei Ansprechen auf die Therapie) sehr schnelle Wirkung zunutze machen möchte, um den Patienten zu retten. Es gibt keine direkten randomisierten Vergleiche, aber Metaanalysen suggerieren, dass, obwohl Patienten unter zielgerichteter Therapie in den ersten 12 Monaten ein besseres Outcome haben, die Immuntherapie wahrscheinlich zu einem besseren Überleben führt [13–15]. Welches die beste Erstlinientherapie ist, wird zurzeit in prospektiven Studien untersucht.

### PD-1-Inhibitoren

Phase-I- und Phase-II-Studien mit den PD-1-Inhibitoren Pembrolizumab [16–18] und Nivolumab [19–21] bei Patienten mit fortgeschrittener Melanomerkrankung zeigten, dass eine signifikante Anzahl von Patienten ein lang anhaltendes progressionsfreies Überleben („progression free survival“ [PFS]) erreichte. Diese Studien waren die Basis für die randomisierten Phase-III-Studien, welche Nivolumab und Pembrolizumab zur Standardtherapie für das fortgeschrittene nichtresektable Melanom machten:

In der CheckMate 066-Studie, einer prospektiven, randomisierten Studie, wurde die Überlegenheit von Nivolumab im Vergleich zur Chemotherapie mit Dacarbazin (DTIC) demonstriert. Nach einem Jahr lag das OS für die Nivolumab Gruppe bei 72,9 % (95 %-KI 65,5–78,9) und in der Dacarbazin Gruppe bei 42,1 % (95 %-KI 33,0–50,9). Die HR für Tod lag in der Dacarbazin Gruppe bei 0,42 (99,79 %-KI 0,25–0,73); *p* < 0,001.

Das mediane PFS betrug 5,1 Monate in der Nivolumab Gruppe versus 2,2 Monate in der Dacarbazin Gruppe. Die HR für Tod oder Progression der Erkrankung lag bei 0,43 (95 %-KI 0,34–0,56; *p* < 0,001) [22]. Die objektive Ansprechrate in der Nivolumab Gruppe betrug 40,0 % (95 %-KI 33,3–47,0) und in der Dacarbazin Gruppe 13,9 % (95 %-KI 9,5–19,4).

Ferner wurde die Überlegenheit der PD-1-Inhibitoren (Nivolumab, Pembrolizumab) gegenüber Ipilimumab in 2 prospektiven, randomisierten Studien, der CheckMate 067 und KEYNOTE 006, gezeigt [23, 24]: In der CheckMate 067-Studie wurde eine HR für Nivolumab vs. Ipilimumab von 0,65 (*p* < 0,001) ermittelt, während die KEYNOTE 006-Studie für Pembrolizumab (10 mg/kg alle 2 Wochen) vs. Ipilimumab eine HR für Tod von 0,63 (*p* < 0,001) und für Pembrolizumab (10 mg/kg alle 3 Wochen) vs. Ipilimumab eine HR für Tod von 0,69 (*p* < 0,001) ermittelte. In Bezug auf das Langzeitüberleben zeigte sich in dieser Studie unabhängig von der Vorbehandlung der Patienten eine 5‑Jahres-OS-Rate von 38,7 % in den gepoolten Pembrolizumab Armen im Vergleich zu 31,0 % im Ipilimumab-Arm. Die 5‑Jahres-OS-Rate bei den therapienaiven Patienten betrug 43,2 % in den gepoolten Pembrolizumab-Armen gegenüber 33,0 % im Ipilimumab-Arm, HR: 0,73 (95 %-KI 0,57–0,92).

Sowohl die Raten der Grad ≥3-Toxizitäten waren vergleichbar hoch zwischen Pembrolizumab und Ipilimumab (17 % vs. 20 %) als auch die Raten des therapieassoziierten Abbruchs (10 % vs. 9 %).

Bezüglich der Therapiedauer bei Patienten, die auf die Therapie ansprechen, gibt es aktuell noch keinen Konsensus und keine Empfehlungen, die in Leitlinien einfließen. Basierend auf Resultaten der KEYNOTE 006-Studie, wird die Empfehlung zur Beendigung der Therapie mit PD-1-Inhibitoren bei Patienten, die auf die Therapie ansprechen (also mindestens eine stabile Erkrankung oder partielle Remission vorweisen) nach 2 Jahren diskutiert. Aufgrund der unten geschilderten Daten kann bei Patienten mit kompletter Remission sogar die Therapiebeendigung nach 6 Monaten diskutiert werden, sobald die komplette Remission nach 2 Stagingrunden bestätigt wird.

Langzeitdaten aus der KEYNOTE 006-Studie zeigten, dass 78,4 % der Patienten, die eine 2‑jährige Therapie mit Pembrolizumab mit mindestens stabiler Erkrankung beenden konnten, nach weiteren 24 Monaten progressionsfrei blieben. Von den 44 Patienten der Studie, die eine komplette Remission erreichten, waren die PFS-Daten ähnlich zwischen den 21 Patienten, die die Therapie 2 Jahre erhielten, und den 23 Patienten, die die Therapie vorher beendeten, aber mindestens 6 Monate durchführten (85 % bzw. 86 % nach 24 Monaten).

Dies sind sicherlich interessante Aspekte der KEYNOTE 006-Studie, für starke Empfehlungen bedarf es allerdings mehrerer Daten.

### Kombinationstherapie mit PD-1-Inhibitor und CTLA-4-Blocker

Zugelassen ist die Kombinationstherapie des CTLA-4-Blockers Ipilimumab in einer Dosierung von 3 mg/kg Körpergewicht intravenös mit Nivolumab 1 mg/kg Körpergewicht intravenös für 4 Zyklen in 3‑wöchentlichem Abstand. Die Therapie wird dann 2‑wöchentlich mit der Nivolumab-Monotherapie fortgeführt.

Die Vorteile der Kombination von Ipilimumab mit Nivolumab im Vergleich zur Monotherapie mit Nivolumab konnten in der prospektiven Phase-III-Studie CheckMate 067 gezeigt werden [23]. In dieser Studie wurde die Kombinationstherapie Ipilimumab/Nivolumab mit der Monotherapie mit Nivolumab und der Monotherapie mit Ipilimumab in therapienaiven Patienten untersucht. In Bezug auf den koprimären Endpunkt PFS zeigte sich eine signifikante Verlängerung in den Nivolumab-Armen im Vergleich zu Ipilimumab. In dem Nivolumab plus Ipilimumab Arm zeigte sich ein medianes PFS von 11,5 Monaten [95 %-KI, 8,9–16,7] vs. einem PFS von 2,9 Monaten [95 %-KI, 2,8–3,4] in dem Ipilimumab Arm (HR für Tod oder Krankheitsprogress: 0,42; [99,5 %-KI, 0,31–0,57]; *p* < 0,001) vs. 6,9 Monaten [95 %-KI, 4,3–9,5] in dem Nivolumab Arm (HR im Vergleich zu Ipilimumab, 0,57; [99,5 %-KI, 0,43–0,76]; *p* < 0,001). Im Oktober 2019 wurden die 5‑Jahres-Daten publiziert [25]: Das mediane OS lag bei mehr als 60 Monaten (Median nicht erreicht) in dem Nivolumab/Ipilimumab-Arm, bei 36,9 Monaten in dem Nivolumab-Arm und bei 19,9 Monaten in dem Ipilimumab-Arm (HR für Tod bei Ipilimumab/Nivolumab vs. Ipilimumab 0,52). Dabei ist nicht zu vergessen, dass Therapien, die Patienten teilweise nachfolgend erhalten haben, ebenfalls für das OS eine Rolle spielen. Das 5‑Jahres-OS lag bei 52 % in dem Kombinationstherapiearm, bei 44 % in dem Nivolumab-Arm und bei 26 % in dem Ipilimumab-Arm. Das Studiendesign erlaubt keinen Vergleich der 2 Nivolumab-enthaltenden Arme aufgrund ungenügender Power. Obwohl alle Endpunkte den Vorteil der Kombinationstherapie hervorheben, bleibt die Verbesserung des OS gering. Es werden Biomarker benötigt, mit denen Patienten selektiert werden können, die von einer Kombinationstherapie profitieren. PD-L1 – ein Biomarker, von dem man sich initial viel versprochen hat, ist beim Melanom von geringem Wert.

Eine andere wichtige Erkenntnis, die aus der CheckMate 067-Studie hervorgeht, bezieht sich auf die Wirkung der Therapie im zeitlichen Verlauf: Komplette Remissionsraten haben stetig in allen Gruppen nach der ersten Publikation zugenommen, was bedeutet, dass sich das beste Ansprechen bei Checkpoint-Inhibitoren mit der Zeit verbessern kann.

Wichtige Entscheidungskriterien bei der Therapiewahl sind das Nebenwirkungsprofil und die Nebenwirkungsrate. Bei betagten, multimorbiden Patienten gilt es, eine effektive Therapie zu finden, die aber auch gut verträglich ist. In der CheckMate 067-Studie zeigten sich hochgradige therapiebedingte Nebenwirkungen (CTC Grad 3 oder 4) bei 16,3 % der Patienten im Nivolumab-Arm, bei 55,0 % im Ipilimumab/Nivolumab-Arm und bei 27,3 % im Ipilimumab-Arm. Aktuell besteht in den meisten Zentren die Tendenz, jungen, sonst gesunden Patienten die Kombinationstherapie anzubieten, während eher eine Monotherapie mit einem PD-1-Inhibitor bei betagten, multimorbiden Patienten bevorzugt wird. Diese Entscheidung ist natürlich patientenindividuell zu treffen. Ausgeprägte Toxizitäten und Therapieabbruch aufgrund von Nebenwirkungen sind häufiger bei der Kombinationstherapie zu beobachten als bei der Monotherapie mit einem PD-1-Inhibitor. Die häufigsten Grad-3/4-Nebenwirkungen waren in der CheckMate 067-Studie gastrointestinale Nebenwirkungen. Die häufigsten Nebenwirkungen jeden Grades waren: Diarrhö, Fatigue, Pruritus und Exantheme. In dem Kombinationstherapiearm kam es bei 39 % der Patienten zum Therapieabbruch aufgrund von Nebenwirkungen (12 % im Nivolumab-Arm und 16 % im Ipilimumab-Arm). Nichtsdestotrotz war ein Therapieabbruch aufgrund von Nebenwirkungen nicht mit einem schlechteren Outcome assoziiert. Es gibt Hinweise, dass eine niedrigere Dosierung von Ipilimumab (1 mg/kg Körpergewicht im Vergleich zur Standarddosis von 3 mg/kg Körpergewicht) zu weniger Toxizitäten führt. Allerdings ist bislang unklar, ob in diesem Fall der Therapieeffekt vergleichbar ist. Hierfür werden weitere Studien benötigt.

Eine spezielle Situation, in der die Kombinationstherapie mit Ipilimumab und Nivolumab als Erstlinientherapie präferiert wird, ist diese des Patienten mit asymptomatischen Hirnmetastasen. In Studien konnten eine Gesamtansprechrate („overall response rate“ [ORR]) von 46 % und ein PFS von 50 % nach 18 Monaten bei Patienten mit asymptomatischen Hirnmetastasen ermittelt werden [26, 27].

Dabei scheinen Patienten mit symptomatischen Hirnmetastasen deutlich schlechter auf die Therapie anzusprechen. Eine weitere Option bei Patienten mit Hirnmetastasen und BRAF-V600-Mutation stellt die zielgerichtete Therapie mit einem BRAF- und MEK-Inhibitor dar. Ansprechraten sind zwar vergleichbar zur Metastasierung in anderen Organen, die Dauer der Antwort ist allerdings verkürzt (PFS mit einem Median von 5,6 Monaten vs. 11 Monaten) [28, 29].

## Ausblick

Es wird erwartet, dass in den nächsten Jahren das Feld der Immuntherapiekombinationen zu den dynamischsten in der Arzneimittelentwicklung gehören wird. ufgrund ihres einzigartigen therapeutischen Index (hohe Effektivität und niedrige Toxizität) spielen PD-1-Inhibitoren eine wichtige Rolle in den zukünftigen Kombinationstherapien. Aktuell gibt es multiple Studien, die diverse Kombinationen untersuchen. So werden aktuell andere Substanzen wie IDO-Inhibitoren, Zytokine, monoklonale Antikörper, Vakzine, zielgerichtete Therapeutika, Chemotherapien, Mikrobiommodulatoren, aber auch die Radiotherapie in Kombination mit PD-1-Inhibitoren untersucht. Es bleibt zu sehen, ob tatsächlich eines Tages von einer Heilung des fortgeschrittenen Melanoms gesprochen werden darf.
